# Supplementary material for: Plasma eNOS Concentration in Healthy Pregnancy and in Hypertensive Disorders of Pregnancy: Evidence of Reduced Concentrations in Pre-Eclampsia from Two Independent Studies
Source: Diseases. 2023 Nov 1;11(4):155. doi: 10.3390/diseases11040155 (PMC10660730; doi:10.3390/diseases11040155)
Supplement: Supplementary file 1 [file diseases-11-00155-s001.zip › diseases-2617240-supplementary.pdf]

Supplementary material

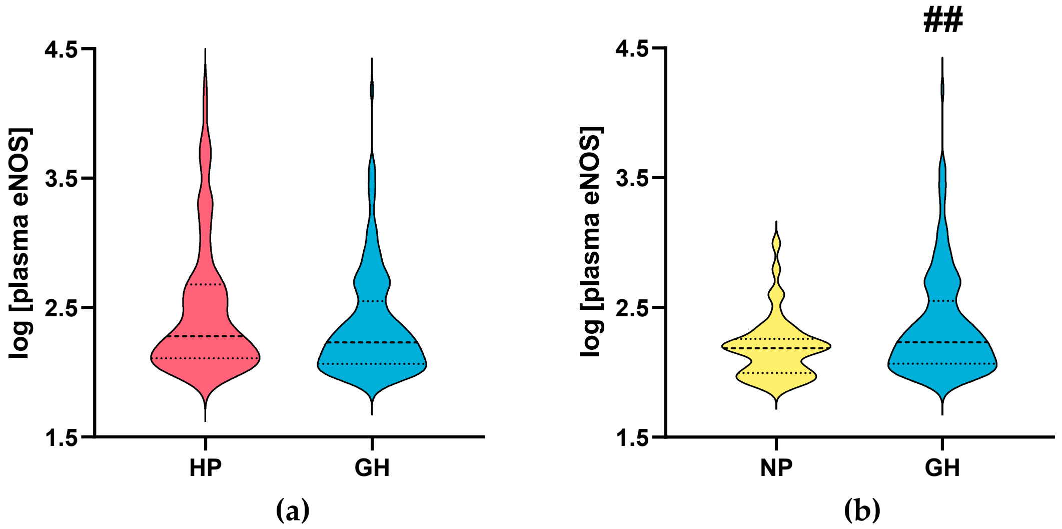

Figure S1. Logarithm of plasma eNOS concentrations from gestational hypertension (GH) patients compared with **(a)** healthy pregnant women (HP), and **(b)** with non-pregnant (NP) women. ## ($p < 0.05$) when compared to NP.

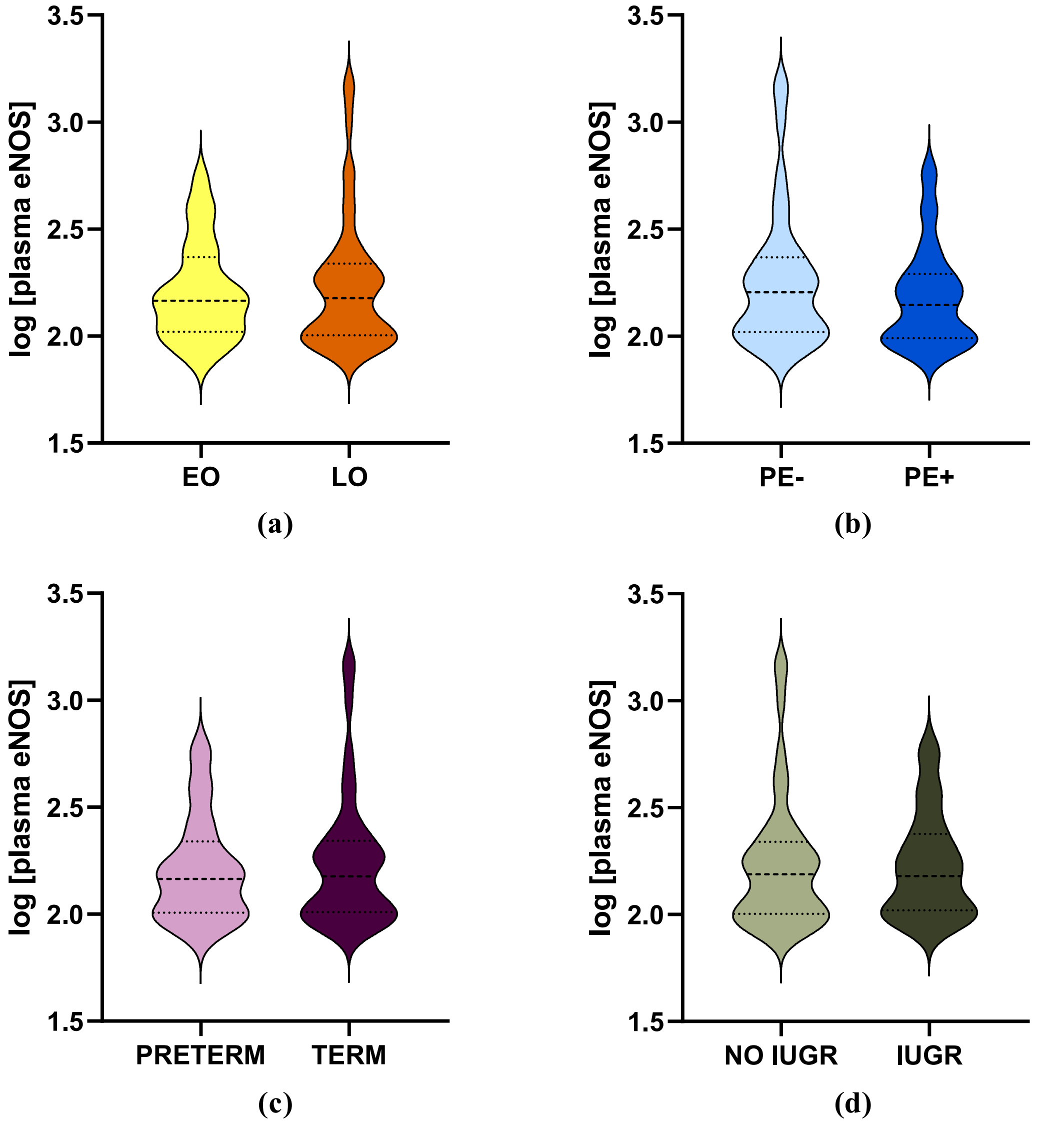

Figure S2. Logarithm of plasma eNOS concentrations considering the subgroups of patients with preeclampsia (PE): **(a)** early-onset PE (EO) and late-onset PE (LO); **(b)**, PE without (PE-) and with (PE+) severe features; **(c)** preterm birth; and **(d)** intrauterine growth restriction (IUGR). None of the comparisons between these groups were significant ($p > 0.05$).

Table S1. Clinical, demographic, and biochemical characteristics of women enrolled in the replication study enrolled in the replication study.

Parameters	Healthy Pregnant (*n* = 41)	Preeclampsia (*n* = 44)	*p*-Value
Age (years)	22.6 ± 4.5	25.9 ± 8.0	0.1238
GAS (weeks)	31.2 ± 5.7	33.9 ± 4.6	**0.0302**
Uric acid (mg/dL)	ND	6.0 ± 1.7	–
24-h Pr (mg/24 h)	NA	2554.0 ± 3782.0	–
Severity (%)	NA	70	–

Abbreviations: GAS, gestational age at sampling; 24-h Pr, 24-h proteinuria; ND, not determined; NA, not applicable. Data are expressed as mean ± S.D. Bold value is significant *p*-value.

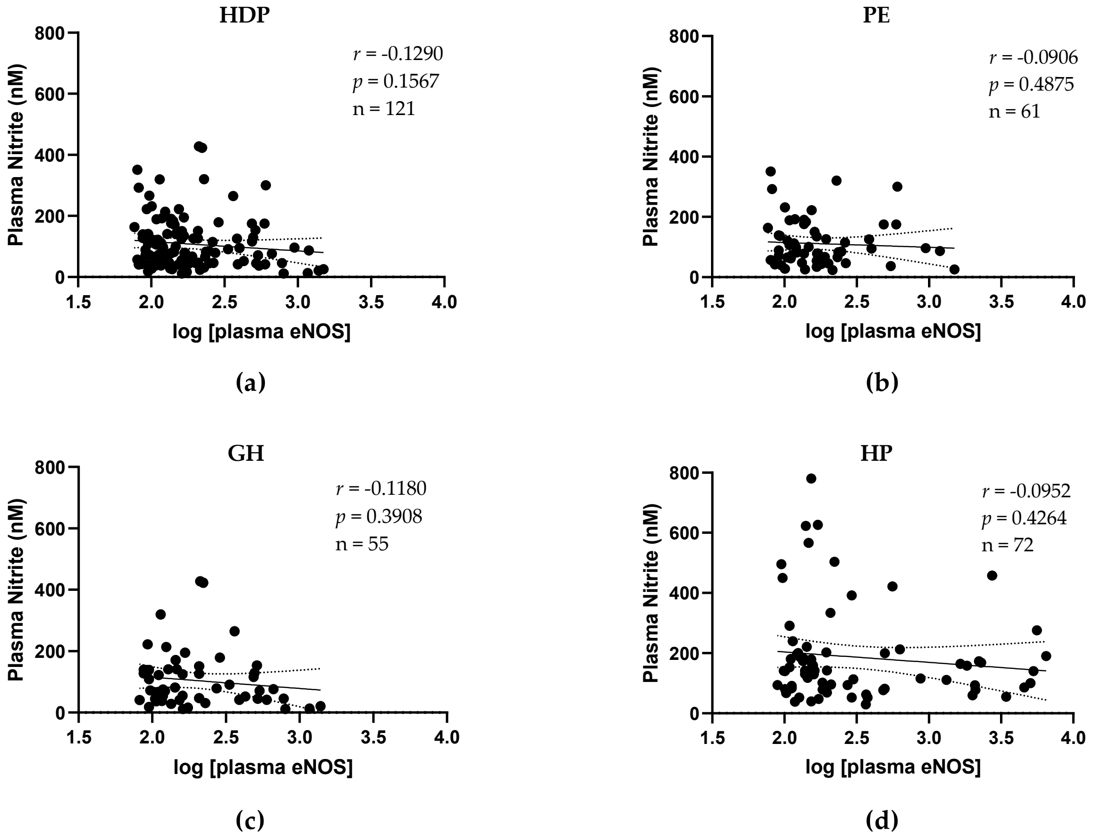

Figure S3. Correlations between logarithm of plasma eNOS concentrations and concentrations of plasma nitrite (nM) in **(a)** women with hypertensive disorders of pregnancy (HDP), pregnant women with **(b)** preeclampsia (PE), **(c)** gestational hypertension (GH), and **(d)** healthy pregnant women (HP).
